# PCR-based detection of *Plasmodium falciparum* in saliva using mitochondrial *cox3* and *varATS* primers

**DOI:** 10.1186/s41182-018-0100-2

**Published:** 2018-06-22

**Authors:** Yukie M. Lloyd, Livo F. Esemu, Jovikka Antallan, Bradley Thomas, Samuel Tassi Yunga, Bekindaka Obase, Nana Christine, Rose G. F. Leke, Richard Culleton, Kenji Obadiah Mfuh, Vivek R. Nerurkar, Diane Wallace Taylor

**Affiliations:** 1 Department of Tropical Medicine, Medical Microbiology and Pharmacology, John A. Burns School of Medicine, University of Hawaii at Manoa, Honolulu, HI 96813 USA; 2 The Biotechnology Center, University of Yaoundé I, Yaoundé, Cameroon; 3 The Faculty of Health Sciences, University of Buea, Buea, Cameroon; 4 Leading Program, Graduate School of Biomedical Sciences, Nagasaki University, Nagasaki, Japan; 5 Malaria Unit, Department of Pathology, Institute of Tropical Medicine (NEKKEN), Nagasaki University, Nagasaki, Japan; 6 Present Address: Cancer Early Detection Advanced Research Center, Knight Cancer Institute, Oregon Health and Science University, Portland, USA

**Keywords:** Malaria diagnosis, Saliva-based assays, Detection, Cameroon, *Plasmodium falciparum*, Malaria surveillance, 18S rRNA, *varATS*, *cox3*, Mitochondrial genome

## Abstract

**Background:**

Sampling of saliva for diagnosing *Plasmodium falciparum* infections is a safe, non-invasive alternative to sampling of blood. However, the use of saliva presents a challenge because lower concentrations of parasite DNA are present in saliva compared to peripheral blood. Therefore, a sensitive method is needed for detection of parasite DNA in saliva. This study utilized two recently reported “ultra-sensitive” PCR assays based on detection of the *P. falciparum* mitochondrial *cox3* gene and the multi-copy nuclear *varATS* gene. The ultra-sensitive assays have an advantage over standard 18S rRNA gene-based PCR assay as they target genes with higher copy numbers per parasite genome. Stored saliva DNA samples from 60 Cameroonian individuals with infections previously confirmed by 18S rRNA gene PCR in peripheral blood were tested with assays targeting the *cox3* and *varATS* genes.

**Results:**

Overall, the standard 18S rRNA gene-based PCR assay detected *P. falciparum* DNA in 62% of the stored saliva DNA samples, whereas 77 and 68% of the samples were positive with assays that target the *cox3* and *varATS* genes, respectively. Interestingly, the ultra-sensitive assays detected more *P. falciparum* infections in stored saliva samples than were originally detected by thick-film microscopy (41/60 = 68%). When stratified by number of parasites in the blood, the *cox3* assay successfully detected more than 90% of infections using saliva when individuals had > 1000 parasites/μl of peripheral blood, but sensitivity was reduced at submicroscopic parasitemia levels. Bands on electrophoresis gels were distinct for the *cox3* assay, whereas faint or non-specific bands were sometimes observed for *varATS* and 18S rRNA that made interpretation of results difficult. Assays could be completed in 3.5 and 3 h for the *cox3* and *varATS* assays, respectively, whereas the 18S rRNA gene assays required at least 7 h.

**Conclusions:**

This study demonstrates that a PCR assay targeting the *cox3* gene detected *P. falciparum* DNA in more saliva samples than primers for the 18S rRNA gene. Non-invasive collection of saliva in combination with the proposed *cox3* primer-based PCR assay could potentially enhance routine testing of *P. falciparum* during disease surveillance, monitoring, and evaluation of interventions for malaria elimination.

**Electronic supplementary material:**

The online version of this article (10.1186/s41182-018-0100-2) contains supplementary material, which is available to authorized users.

## Background

Early and accurate diagnosis of *Plasmodium falciparum* (*Pf*) infections is crucial for treatment and monitoring of malaria transmission. Thick-film microscopy (TFM) has traditionally been the gold standard for the identification of *Pf* from peripheral blood. A trained microscopist can typically detect 50–100 parasites/μl of blood in Giemsa’s solution-stained blood smear [1], but individuals with fewer parasites may be erroneously diagnosed as parasite-free. Although people with submicroscopic infections are usually not ill, they are capable of transmitting sexual-stage parasites to mosquitoes [2]. Submicroscopic infections can be detected by highly sensitive molecular assays, such as nested PCR (nPCR) and loop-mediated isothermal amplification (LAMP) [3]. With the use of nPCR and LAMP, the limit of detection is estimated to be 0.1–10 parasites/μl of blood and can be lower still with qRT-PCR [4]. Current diagnostic and molecular methods have an inherent problem; they require collection of peripheral blood. Routine blood collection methods are invasive procedures that are painful to patients, require trained phlebotomists, and involve the use of needles or lancets. Improper handling of sharps exposes healthcare workers and the patient to blood-borne pathogens, while disposal of used sharps is hazardous for waste management in malaria-endemic countries [5]. Repetitive sampling of blood from the same person, e.g., in mass studies monitoring malaria elimination, often results in low compliance.

Non-blood samples, e.g., saliva and urine, can serve as non-invasive and safe alternatives to blood samples. *Plasmodium* DNA has been detected in saliva and urine [6], however, at 600 and 2500 times lower levels, respectively [7], than those present in peripheral blood. Despite the reduced amount of parasite DNA in saliva, quantitative studies show a positive correlation between parasite numbers detected in peripheral blood by microscopy and parasite DNA in saliva [7, 8], which supports the use of saliva as an alternative for blood.

The traditional molecular assay used for *Pf* detection targets a specific region in the 18S ribosomal RNA (18S rRNA) gene [9] that has 4–8 copies per *Pf* genome. Recently, primers to multi-copy gene targets have been reported to have improved sensitivity. One is the mitochondrial cytochrome c oxidase III (*cox3*) gene, with 20 to 150 copies per *Pf* genome. Because *cox3* is a mitochondrial gene, it is less likely to undergo immune pressure and genetic variation. The other gene is the *var* gene acidic terminal sequence (*varATS*), ~ 59 copies/*Pf* genome [4, 10, 11]. Cunha et al. reported detection of submicroscopic infections in the blood by qPCR using *varATS* primers that were missed by 18S rRNA gene primers. Since conventional instrumentation of qPCR can be difficult to maintain in resource poor countries where malaria is transmitted, a conventional PCR method might be more useful.

A recent study was conducted in Cameroon using 18S rRNA gene PCR to detect *Pf* DNA in the saliva of 222 fever patients. The study was able to detect *Pf* DNA in saliva in 95% [95% CI 85–99] of 53 subjects who were peripheral blood smear positive by microscopy and 82% [95% CI 72–90] of 78 blood samples positive by PCR [8]. The current study hypothesized that the use of these “ultra-sensitive” primers to detect malaria DNA in saliva would increase the number of *Pf* cases detected compared to the use of the traditional 18S rRNA gene PCR.

## Methods

### Sample selection

Paired blood and saliva samples were collected in 2015 from febrile patients in Cameroon as previously described [8]. Saliva were collected using the OMNIgene®ORAL (OM-501) kits (Genotek, Ottawa, Canada). In the original study, DNA was isolated from whole blood and saliva using a DNA purification kit (Macherey Nagel, Duren, Germany) and stored at − 20 °C until used. In the current study, 60 stored archival saliva DNA samples were available from subjects who originally tested peripheral blood positive by PCR using 18S rRNA gene PCR. As a positive control, DNA isolated from laboratory-cultured *Pf* of the 3D7 strain was serially diluted five-folds from 3.74 to 4 × 10^−7^ ng/μl of parasite DNA. Negative controls were saliva samples collected from three U.S. individuals who were malaria naïve, and a non-template control with nuclease-free PCR-grade water was substituted for the DNA template.

### Nested PCR amplification of 18S rRNA

In the original study, the following protocol was used. One PCR reaction consisted of 12.5 μl of 2x Go Taq Green (Promega, Madison, USA), 1 μl each of 10 μM forward and reverse primers (IDT, IA, USA), 5.5 μl of nuclease-free water, and 5 μl of DNA template, with a total of 25 μl. The standard protocols for the cycling conditions were followed [9]. For the initial amplification targeting the 18S rRNA gene, forward and reverse primers, rPLU5 (5′-CCTGTTGTTGCCTTAAACTTC-3′) and rPLU6 (5′-TTAAAATTGTTGCAGTTAAAACG-3′) were used. Five microliters of the amplified product was used for the second round of amplification with forward and reverse primers, rFAL-1 (5′-TTAAACTGGTTTGGGAAAACCAAATATATT-3′) and rFAL-2 (5′-ACACAATGAACTCAATCATGACTACCCGTC) [9]. The expected product size was 205 base pairs (bp). In the current study, the above protocol was employed, except 5 μl of DNA was used (instead of 2 μl) to allow for direct comparison among the primers.

### Nested PCR amplification of mitochondrial cytochrome C oxidase III (*cox3*)

For the initial amplification targeting *cox3*, forward and reverse primers, MtU.F (5′-CTCGCCATTTGATAGCGGTTAACC-3′) and MtU.R (5′-CCTGTTATCCCCGGCGAACCTTC-3′) were used with standard protocols for the cycling conditions [10]. Five microliters of the amplified product, diluted at 1:50, was used for the second amplification with forward and reverse primers MtNst_falF (5′-GAACACAATTGTCTATTCGTACAATTATTC-3′) and MtNst_falR (5′-CTTCTACCGAATGGTTTATAAATTCTTTC-3′). The expected product size was 201 bp.

### PCR amplification of varATS

For the amplification targeting *varATS*, forward *varATS* primer (5′-CCCATACACAACCAAYTGGA-3′) and reverse *varATS* primer (5′-TTCGCACATATCTCTATGTCTATCT-3′) were used with standard protocols for the cycling conditions [4]. The expected product size was 65 bp.

### Gel electrophoresis

Ten microliters of the PCR products were run on a 1.5% agarose gel (Sigma, Fisher, USA) stained with ethidium bromide (Sigma-Aldrich, USA) for 45 min at 100 V. Products were visualized under a UV lamp. A 100-bp ladder (exACTGene Ladder, Fisher) was used as a reference. The presence of a band at the expected size for each primer set was determined positive.

### Statistical data analysis

Data analyses were conducted using Microsoft Excel and Prism version 7.04 (Graph Pad Software Inc.). The 95% confidence interval was calculated using the modified Wald method.

## Results

### Comparison of the limit of detection of the three primers

DNA isolated from *P. falciparum* cultured in vitro that contained from 3.74 to 4 × 10^−7^ ng/μl of parasite DNA was serially diluted fivefold and used as the positive control in the assays. The lower limit of detection (i.e., presence of a band following gel electrophoresis) using the 18S rRNA primers reached 1 × 10^−5^ ng/μl; *cox3* primer was 4 × 10^−7^ ng/μl; and *varATS* primer was 2 × 10^−6^ ng/μl. It is important to point out that both the 18S rRNA and *cox3* assays are nested PCRs, where 5 μl of the nest 1 produce is used in nest 2 of the 18S rRNA assay, but 5 μl of a 1:50 dilution of the nest 1 was used in nest 2 of the *cox3* assay. The *varATS* assay was a single-step PCR assay. Thus, the *cox3* assay was the most sensitive assay, with the *varATS* having a lower limit of detection similar to that of the nest-PCR 18S rRNA method.

### Detection of *Pf* DNA in saliva using 18S rRNA primers, cox3, and varATS

Overall, the PCR assay using standard 18S rRNA primers detected *Pf* DNA in 62% [95% CI 49–73] of the stored saliva DNA samples, whereas 77% [95% CI 64–86] and 68% [95% CI 56–79] of the samples were positive with assays using *cox3* and *varATS* primers, respectively (Table 1). Although the differences were not significant based on 95% CI, the *cox3* primers detected more submicroscopic infections compared to the 18S rRNA and *varATS* (47% vs. 21 and 26%, respectively) and had the highest efficiency for malaria detection (Table 2). Interestingly, the ultra-sensitive primers detected *Pf* in more stored saliva DNA samples than were originally detected by TFM (41/60 = 68%) and thus were more efficient (Table 2). When stratified by number of parasites in the blood, the *cox3* assay detected over 90% of infections using saliva DNA when individuals had > 1000 parasites/μl in their peripheral blood (Table 1). For individuals with > 10,000 parasites/μl in peripheral blood, both *cox3* and *varATS* assays successfully detected 100% of the infections, but the sensitivity was reduced at submicroscopic parasitemia levels. US control samples were negative for all primer sets (Additional file 1: Table S1).

**Table 1:** Percentage of saliva samples that tested positive for *P. falciparum* by parasitemia level (*n* = 60 samples)

		Number positive/number tested (% positive)		
Parasites/μl	*n*	18S rRNA	*cox3*	*varATS*
Submicroscopic	19*	4/19 (21)	9/19 (47)	5/19 (26)
< 1000	6	3/6 (50)	3/6 (50)	4/6 (67)
> 1000	35	30/35 (86)	34/35 (97)	29/35 (83)
All samples	60	62 [49–73] (37/60)	77 [64–86] (46/60)	68 [56–79] (41/60)

All samples: data in square brackets show 95% CI calculated using modified Wald method

All samples: data in parentheses show the number of samples that tested positive/total

*Peripheral blood samples from these individuals were PCR positive for malaria

**Table 2:** Comparison of the efficiency of thick-film microscopy and PCR

		Reference technique			
		Microscopy blood (2015)	18S rRNA PCR saliva	*cox3* PCR saliva	*varATS* PCR saliva
Experimental technique*	Microscopy blood (2015)	1.00			
	18S rRNA PCR saliva	0.9	1.00		
	cox3 PCR saliva	1.12	1.24	1.00	
	varATS PCR saliva	1.00	1.11	0.89	1.00

*Values greater than 1 indicate higher efficiency than the reference technique. Values less than 1 indicate lower efficiency than reference technique

### Advantages and disadvantages of each assay

**Fig. 1:**
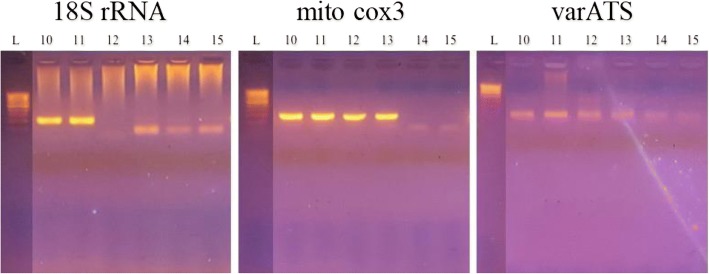
Representative photographs of gel electrophoresis for 18S rRNA, *cox3*, and *varATS* primers using six saliva DNA samples (sample nos. 10–15). Sample nos. 10 and 11 were positive with 18S rRNA, since distinct bands of the correct size were visible; sample nos. 10 through 13 were positive with *cox3*; and sample nos. 10 and 11 were positive with *varATS* primers, but sample nos. 12–15 were difficult to score. The gel pictures show brighter and more distinct bands for *cox3* than 18s rRNA and *varATS*

*Cox3* PCR consistently showed the brightest and sharpest bands on the gel, whereas the 18S rRNA PCR often produced smeared and non-specific bands with a different size from the expected size (Fig. 1). Interpretation of the gel was also difficult for *varATS* PCR, as bands were constantly faint and resulted in over 10% of “unclear” samples (Additional file 1: Table S1). The ultra-sensitive primers-based PCR had another advantage over traditional PCR with much shorter turnaround times than 18S rRNA, ~ 3.5 and ~ 3 h for *cox3* and *varATS*, respectively, compared to ~ 7 h for 18S rRNA (Table 3).

**Table 3:** Comparison of turnaround time for PCR and gel electrophoresis

	18S rRNA	*cox3*	*varATS*
Nest 1	2 h 40 min	1 h 25 min	2 h 8 min
Nest 2	3 h 15 min	59 min	n/a
Gel	45 min	45 min	45 min
Total	~ 7 h	~ 3.5 h	~ 3 h

*n/a* not applicable

## Discussion

Non-invasive and sensitive tests for malaria have many applications. This study compared the use of three PCR-based assays to detect *Pf* DNA in saliva. Saliva samples have multiple advantages over blood samples, e.g., no pain for patients, minimal training required for healthcare workers, reduced transmission of blood-borne pathogens, and contaminated sharp wastes. The WHO reported that 37.6% of hepatitis B, 39% of hepatitis C, and 4.4% of HIV/AIDS prevalence in health workers worldwide are due to needle-stick injury [12]. Saliva tests have been shown to be convenient and safe for cancer, HIV, HCV, HPV, and recently in the field of malaria by detecting HRP-2, lactate dehydrogenase, and *Pf* DNA [6, 13–18].

In the current study, two sets of primers were evaluated, namely primers to the *P. falciparum* genes for *cox3* and *varATS*, which were first described in 2015. Following its initial report, the *cox3* PCR has been used in epidemiological studies [19, 20]. Since the assay is a nested PCR, *cox3* PCR has the benefit of identifying the presence or absence of *Plasmodium* species in the blood after a single round of PCR amplification. The mitochondrial genome is highly conserved in *Plasmodium* species [21]. *Plasmodium* detection using another mitochondrial gene, cytochrome b gene (*cytb*), in saliva and urine has been reported in symptomatic patients [6]. However, a more recent study demonstrated that *cytb*-based PCR has lower sensitivity than *cox3*-based assays [10].

In a resource- and personnel-limited setting, clear and easy interpretation of results is extremely important and the presence of a single distinct band in gel electrophoresis-based assays help assure accurate diagnosis. The traditional 18S rRNA gene-based PCR frequently amplified non-specific bands (Fig. 1), a phenomenon also been reported by others [10, 22]. The extra bands could possibly be due to cross reactivity between human and parasite small subunit rRNA [10]. In contrast, *cox3* primers target a gene specific to *Plasmodium*, thereby eliminating the risk of non-specific amplification of human DNA. *VarATS* PCR uses a single amplification and produces a small-sized product, making it difficult for researchers to be confident in distinguishing between the true product and non-specific bands on the gel. On the other hand, *varATS* primers have the advantage of being used in a single-step PCR, thereby significantly reducing the turnaround time and workload required for nPCR.

The process of how parasite DNA enters the human salivary glands and circulates in the saliva remains unclear. Further investigation is also required to determine whether *Pf* DNA in the saliva is present only during an active *Pf* infection or if it persists after parasites are cleared from the blood. An inherent problem with rapid diagnostic test based on detection of parasite histidine-rich protein-two (HRP-2) is the persistence of the antigen in the peripheral blood after parasite clearance [23–25].

The project has several limitations. The DNA samples were collected in 2015 and DNA was isolated and stored at − 20 °C until re-evaluated in 2017. The saliva samples were collected in 2015 and DNA degradation was likely to have occurred, as the detection of positive saliva samples was higher in the original study than that when archival saliva DNA was used in 2017 with the 18S rRNA primers. DNA degradation in stored samples is not unexpected, as power outages frequently occur in Cameroon. Whether DNA degradation took place is not clear, but it is likely that ultra-sensitive primers would perform even better using fresh saliva samples.

In future studies, a larger number of fresh saliva and blood should be collected and tested with 18S rRNA, *cox3*, and *varATS* primers to establish the true sensitivity of each PCR-based approach. In addition, future studies are needed to determine if other species of malaria (e.g., *P. vivax, P. malariae*, *P. ovale)* can also be diagnosed using saliva and if the amount of Plasmodial DNA in saliva reflects the level of parasitemia in the peripheral blood.

## Conclusions

The present study found the PCR assay based on detection of the mitochondrial *cox3* gene to be better than the traditional 18s rRNA assay for detecting *Pf* DNA in stored DNA samples extracted. In addition, PCR using *cox3* primers provided bright and distinct band following gel electrophoresis and the assay could be completed in ~ 3.5 h. The proposed combination of saliva and PCR using *cox3* primers may provide a new tool for use in diagnosis, epidemiological studies, and monitoring interventions for malaria control.

## Additional file

Additional file 1: **Table S1.** Raw data are provided in the Table. The results have been sorted by parasitemia that was determined by thick-film microscopy. Shaded blocks indicate where a difference between the three PCR assays evaluated was seen. (DOCX 26 kb)

## Abbreviations

18S rRNA: 18S small subunit ribosomal RNA
AIDS: Acquired Immune Deficiency Syndrome
bp: Base pair
*cox3*: Mitochondrial cytochrome c oxidase III
Cytb: Cytochrome b gene
HIV: Human immunodeficiency virus
HPV: Human papillomavirus
HRP-2: Histidine-rich protein 2
LAMP: Loop-mediated isothermal amplification
nPCR: Nested polymerase chain reaction
*Pf*: *Plasmodium falciparum* or *P. falciparum*
qPCR: Qualitative PCR
qRT-PCR: Quantitative reverse-transcription PCR
TFM: Thick-film microscopy
*varATS*: var gene acidic terminal sequence
WHO: World Health Organization
