# Supplementary material for: PCR-based detection of Plasmodium falciparum in saliva using mitochondrial cox3 and varATS primers
Source: Trop Med Health. 2018 Jun 22;46:22. doi: 10.1186/s41182-018-0100-2 (PMC6013985; doi:10.1186/s41182-018-0100-2)
Supplement: Supplementary file 1 — Table S1. Raw data are provided in the Table. The results have been sorted by parasitemia that was determined by thick-film microscopy. Shaded blocks indicate where a difference between the three PCR assays evaluated was seen. (DOCX 26 kb) [file 41182_2018_100_MOESM1_ESM.docx]

Table S1: Raw data are provided in the Table. The results have been sorted by parasitaemia that was determined by thick film microscopy. Shaded blocks indicate where a difference between the 3 PCR assays evaluated were seen.

| ID # | Age | BLOOD -2015 | | |  | SALIVA -2017 | | |
| --- | --- | --- | --- | --- | --- | --- | --- | --- |
|  |  | Microscopy | Parasitaemia* | RDT |  | 18S rRNA | cox3 | varATS |
| 1 | 78 | Neg | 0 | Neg |  | Neg | Neg | Neg |
| 2 | 26 | Neg | 0 | Neg |  | Neg | Pos | ? |
| 4 | 5 | Neg | 0 | Pos |  | Neg | Pos | Neg |
| 5 | 28 | Neg | 0 | Neg |  | Neg | Neg | Neg |
| 6 | 21 | Neg | 0 | Neg |  | Neg | Neg | ? |
| 7 | 23 | Neg | 0 | Pos |  | Pos | Pos | Pos |
| 8 | 25 | Neg | 0 | Neg |  | Neg | Neg | Neg |
| 12 | 20 | Neg | 0 | Neg |  | Neg | Pos | ? |
| 25 | 19 | Neg | 0 | Neg |  | Neg | Pos | Pos |
| 28 | 6 | Neg | 0 | Neg |  | Neg | Neg | Neg |
| 29 | 11 | Neg | 0 | Neg |  | Neg | Neg | Neg |
| 39 | 9 | Neg | 0 | Pos |  | Pos | Neg | ? |
| 42 | 4 | Neg | 0 | Pos |  | Neg | Neg | ? |
| 46 | 30 | Neg | 0 | Pos |  | Neg | Pos | Pos |
| 48 | 7 | Neg | 0 | Pos |  | Pos | Pos | Pos |
| 50 | 9 | Neg | 0 | Pos |  | Neg | Neg | Pos |
| 52 | 5 | Neg | 0 | Pos |  | Neg | Pos | ? |
| 56 | 4 | Neg | 0 | Pos |  | Pos | Pos | ? |
| 58 | 36 | Neg | 0 | Neg |  | Neg | Neg | Neg |
| 57 | 30 | Pos | 80 | Pos |  | Neg | Neg | Neg |
| 37 | 10 | Pos | 120 | Pos |  | Pos | Pos | Pos |
| 53 | 73 | Pos | 120 | Pos |  | Neg | Pos | Pos |
| 13 | 22 | Pos | 320 | Pos |  | Neg | Neg | Neg |
| 43 | 23 | Pos | 800 | Pos |  | Pos | Pos | Pos |
| 35 | 58 | Pos | 1000 | Pos |  | Pos | Pos | Pos |
| 14 | 7 | Pos | 1400 | Pos |  | Neg | Neg | Neg |
| 45 | 20 | Pos | 1880 | Pos |  | Pos | Neg | Pos |
| 33 | 7 | Pos | 1960 | Pos |  | Pos | Pos | Pos |
| 12 | 19 | Pos | 2200 | Pos |  | Neg | Pos | ? |
| 11 | 16 | Pos | 2760 | Pos |  | Pos | Pos | Pos |
| 31 | 28 | Pos | 2920 | Pos |  | Pos | Pos | Pos |
| 59 | 9 | Pos | 5160 | Pos |  | Pos | Pos | Pos |
| 55 | 11 | Pos | 8900 | Pos |  | Pos | Pos | Pos |
| 20 | 7 | Pos | 9120 | Pos |  | Neg | Pos | Neg |
| 34 | 28 | Pos | 9720 | Pos |  | Pos | Pos | Pos |
| 18 | 32 | Pos | 12840 | Pos |  | Pos | Pos | Pos |
| 38 | 40 | Pos | 13600 | Pos |  | Pos | Pos | Pos |
| 32 | 13 | Pos | 20800 | Pos |  | Pos | Pos | Pos |
| 60 | 13 | Pos | 25714 | Pos |  | Pos | Pos | Pos |
| 17 | 22 | Pos | 26760 | Pos |  | Neg | Pos | Pos |
| 30 | 22 | Pos | 27000 | Pos |  | Pos | Pos | Pos |
| 51 | 51 | Pos | 32160 | Pos |  | Pos | Pos | Pos |
| 40 | 32 | Pos | 40160 | Pos |  | Pos | Pos | Pos |
| 19 | 15 | Pos | 43680 | Pos |  | Pos | Pos | Pos |
| 9 | 18 | Pos | 49600 | Pos |  | Pos | Pos | Pos |
| 54 | 18 | Pos | 57120 | Pos |  | Pos | Pos | Pos |
| 27 | 20 | Pos | 59520 | Pos |  | Pos | Pos | Pos |
| 26 | 30 | Pos | 60285 | Pos |  | Pos | Pos | Pos |
| 15 | 8 | Pos | 67200 | Pos |  | Pos | Pos | Pos |
| 36 | 5 | Pos | 76615 | Pos |  | Pos | Pos | Pos |
| 47 | 7 | Pos | 77440 | Pos |  | Pos | Pos | Pos |
| 22 | 27 | Pos | 78400 | Pos |  | Pos | Pos | Pos |
| 23 | 20 | Pos | 99680 | Pos |  | Pos | Pos | Pos |
| 3 | 23 | Pos | 109714 | Pos |  | Pos | Pos | Pos |
| 10 | 6 | Pos | 123360 | Pos |  | Pos | Pos | Pos |
| 21 | 10 | Pos | 158240 | Pos |  | Pos | Pos | Pos |
| 41 | 5 | Pos | 177230 | Pos |  | Pos | Pos | Pos |
| 24 | 13 | Pos | 314285 | Pos |  | Pos | Pos | Pos |
| 49 | 33 | Pos | 498285 | Pos |  | Neg | Pos | Pos |
| 44 | 5 | Pos | 587428 | Pos |  | Pos | Pos | Pos |
|  |  |  |  |  |  |  |  |  |
| * 0 = submicroscopic infections; # infected erythrocytes / ul | | | | | | | | |
| ? Difficult to read, no definitive answer. Note: 7 of the 8 difficult to read samples | | | | | | | | |
|  | had submicroscpic infections | | | | | | | |
|  | Highlighted areas indicate disagreement between the 3 assays | | | | | | |  |
|  |  |  |  |  |  |  |  |  |
| USA Negative controls | | |  |  |  |  |  |  |
| C1 |  | N/A | N/A | N/A |  | Neg | Neg | Neg |
| C2 |  | N/A | N/A | N/A |  | Neg | Neg | Neg |
| C3 |  | N/A | N/A | N/A |  | Neg | Neg | Neg |
